# Fluctuating hypermetropia due to intraocular lens displacement caused by iris pigment epithelial cyst

**DOI:** 10.1016/j.ajoc.2024.102065

**Published:** 2024-05-03

**Authors:** Kirupakaran Arun, Nizar Din, Miles Stanford, Victoria Cosgrove, Mukhtar Bizrah

**Affiliations:** a Imperial College Healthcare NHS Trust, Western Eye Hospital, 153-173 Marylebone Road, London, NW1 5QH, United Kingdom; b Harley Vision, St John & St Elizabeth Hospital, 60 Grove End Rd, London, United Kingdom

**Keywords:** Hyperopic shift, Refractive error, Refractive surprise, Iris pigment epithelial cyst, Intraocular lens displacement

## Abstract

**Purpose:**

To report a case of hyperopic shift following lens replacement surgery due to an enlarging iris pigment epithelial (IPE) cyst.

**Observations:**

A gentleman presented with reduced visual acuity (Snellen unaided 20/25) 12 months followed lens replacement surgery. Examination revealed a retro-pupillary iris lesion that appeared to be displacing the posterior chamber intraocular lens (IOL) and was causing a hyperopic shift (refraction +2.00). Anterior segment optical coherence tomography imaging confirmed this to be an IPE cyst with a posteriorly displaced IOL body. After observation over 30 months, the IPE cyst spontaneously reduced in size and the IOL returned to a more physiological position. Unaided visual acuity improved to Snellen 20/16 and refraction improved to +0.50.

**Conclusions and Importance:**

To our knowledge, an IPE cyst that shows growth following intraocular surgery has not previously been reported. This growth resulted in a hyperopic shift due to posterior displacement of the IOL. This case demonstrates spontaneous regression of the cyst, and suggests that over time these cysts can change in size.

## 1 Introduction

Primary iris pigment epithelial (IPE) cysts commonly are asymptomatic and are stationary or very slow-growing.^1^ Secondary iris cysts are less common but are more prone to causing complications including reduced vision, glaucoma and uveitis.^2^ We report a unique case of a gentleman who presented with posterior intraocular lens (IOL) displacement due to a large IPE cyst behind the iris.

## 2 Case report

**Fig. 1:**
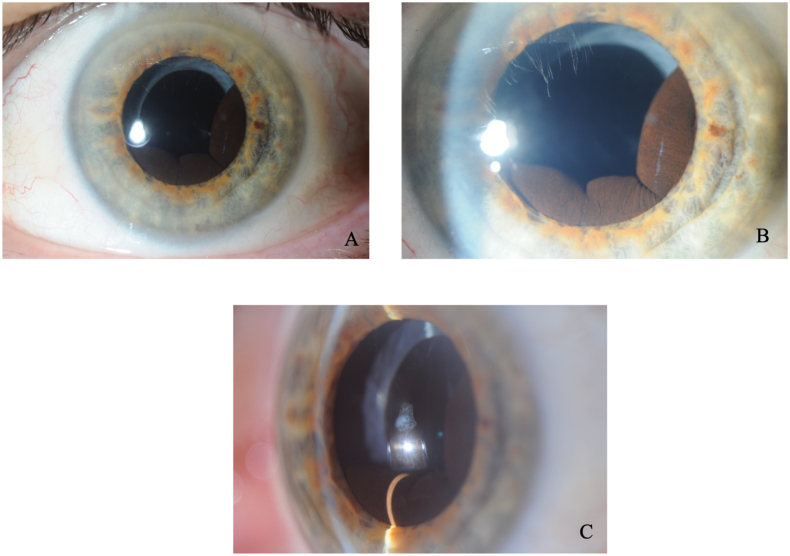
(A): Slit lamp photograph (low magnification) of the left eye showing a retro-pupillary iris lesion through dilated pupil. (B): Slit lamp photograph (high magnification) of same lesion through dilated pupil (C): Slit lamp photograph showing iris lesion protruding posterior to pupillary plane.

A 66-year-old male presented to the outpatient department due to blurred vision in his left eye following lens replacement surgery with a standard monofocal intraocular lens. He had a past ocular history of bilateral LASIK (laser-assisted in situ keratomileusis) for hypermetropia and more recent bilateral lens replacement surgery. On examination, the Snellen unaided visual acuity was 20/20 and 20/40, respectively on the right and left eyes. Left eye vision improved to 20/20 with pinhole. Autorefraction was +0.25/-1.00 × 35 on the right eye and +2.00/0.00 × 180 on the left eye. Intraocular pressures were normal in both eyes. The right eye anterior and posterior examination was unremarkable. However, the left examination revealed a clear cornea, quiet anterior chamber with a retro-pupillary iris mass lesion from 2 to 9 o clock (Fig. 1 A, B and C). Posterior segment examination of the left eye was normal.

**Fig. 2:**
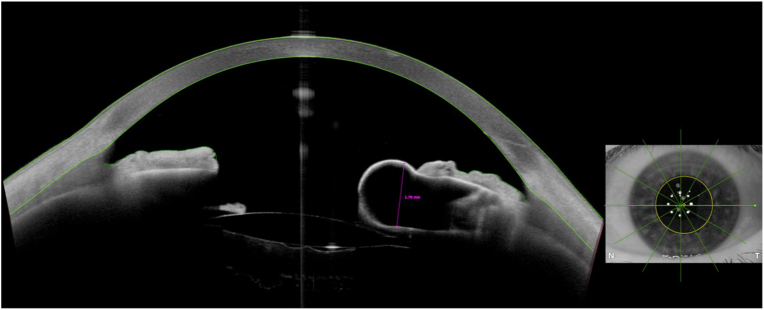
Anterior segment optical coherence tomography images of the left eye showing a typical retro-pupillary iris cyst (1.78mm thickness).

High-resolution anterior segment optical coherent tomography (AS-OCT) imaging of the left eye further demonstrated this lesion. It measured 1.78mm in thickness with internal lucency and was seen protruding from the posterior iris and causing posterior displacement of the posterior chamber IOL (Fig. 2).

A diagnosis of left midzonal IPE cyst was made. This was causing a hyperopic shift from posterior displacement of the posterior chamber IOL. On further questioning, the patient did recollect being told at the time of his left lens replacement surgery that there was a small iris cyst, but this did not affect surgery in any way.

With regards to treatment options, the patient enquired about further laser refractive surgery to correct the hypermetropia. Due to the seemingly sudden increase in size of the iris cysts post lens replacement surgery, we recommended spectacle/contact lens to correct the hypermetropia in the first instance and arranged a further review in the clinic to monitor the cyst.

Four months later, the patient attended for a follow-up appointment. Unaided vision in the left eye had spontaneously improved to Snellen 20/25 and refraction had improved to +1.25/0.00 × 180. On examination, the left iris pigment epithelial cyst had become visibly smaller. As the cyst had become spontaneously smaller and unaided vision and refraction had improved, we decided on surveillance monitoring over any intervention to remove the cyst.

The patient was reviewed again twelve months following the initial presentation. The iris cyst had decreased in size. Vision in the left eye had further improved to Snellen 20/16 unaided and refraction had improved to +0.75/-0.50 × 144.

Humphrey visual fields remained full in both eyes with normal intraocular pressures and healthy optic nerves. Over 2.5 years later following initial presentation, his visual acuity remains Snellen 20/16 unaided with plano refraction.

## 3 Discussion

Iris cysts are uncommon and can be classified as primary or secondary according to aetiology, and then further divided into subgroups based on their tissue or origin, with primary IPE cysts representing 86 % of all iris cysts.^4^

Primary IPE cysts are thought to be remnants of the marginal sinus that have not been obliterated and represent developmental anomalies resulting from the cystic expansion of the potential space between the pigmental epithelial layers of the posterior iris.^3^ Histopathology of primary IPE cysts is composed of nonkeratinized squamous epithelial-lined structures that arise from the posterior iris layer.^5^

Primary IPE cysts are usually an incidental finding. Primary IPE cysts can be classified based on their location: pupillary, midzone, peripheral and free-floating/dislodged.^6^ In terms of incidence, peripheral IPE cysts are most common (64 %), followed by midzone (28 %), pupillary (7 %) and dislodged (1 %).^7^

The clinical course of primary IPE cysts is dependent on their location.^1^ Peripheral, midzone and dislodged IPE cysts tend to remain stable, regress in size or completely resolve. However, pupillary IPE cysts are more likely to enlarge over time (84 %), with the remainder tending to remain stable in size (8 %) or completely resolve (8 %).^7^ Interestingly, our patient had a midzone IPE cyst that did show initial enlargement, followed by regression.

Despite this, in the largest primary IPE surveillance study conducted, no complications were detected with pupillary IPE cysts.^7^ Instead, complications were more seen with midzone and peripheral IPE cysts and these included corneal touch, focal cataract, lens subluxation, iritis and glaucoma.^7^

Imaging modalities such as ultrasound biomicroscopy and AS-OCT are essential in differentiating iris cysts from ocular tumours. IPE cysts appear as thin-walled and homogeneous lesions with hypoechoic internal content and regular borders on UBM and AS-OCT,^8^ whilst tumours present with a solid inner structure^9^ and show increase in lesion size.^10^ Based on the history, examination and AS-OCT, our patient had a left midzonal primary IPE cyst.

It seems that there was transient enlargement of the IPE cyst in our patient following lens replacement surgery, and this caused the IOL body to displace posteriorly and produce a hyperopic shift (he was emmetropic after his lens extraction). The lack of astigmatism change is somewhat peculiar as the asymmetrical pressure on the IOL body from the transient IPE cyst enlargement would be expected to produce some change in astigmatism. On examination, the IOL was well-centred. There was evidence of posterior displacement, but no evidence of decentration or tilt. This would explain the change in spherical refractive error, but no induction of cylindrical error.

There is clear evidence that trauma,^2^ inflammation^1^ and even certain glaucoma medication such as latanoprost^11^ can stimulate the formation of secondary iris cysts. However, the possible pathogenic mechanism underlying enlargement of primary IPE cysts is still unclear and there are no reports of enlargement/reduction in size of primary IPE cysts being linked to intraocular surgery.^12^ Furthermore, the two largest IPE surveillance studies^7^^,^^8^ did not identify intraocular surgery as a risk factor for cyst enlargement. There are reports of secondary iris cysts (following lens extraction surgery) growing and causing the IOL body to become posteriorly displaced with subsequent hyperopic shift.^13^, ^14^, ^15^

There is very limited literature to show that primary iris cysts have any effect on posterior chamber “in-the-bag” IOLs as our case showed. However, there is some data on how iris cysts affect the position of implantable collamer lenses (ICLs). One study reported a case series of 218 eyes with iris cysts and found no abnormal effects on ICL position, vault or astigmatism over 12 months.^16^ Another study showed no difference in the ICL position, central vault of ICL and refractive error following ICL implantation between eyes with and without primary iris cysts.^17^ Similar to what occurred in our case, both these studies found that most cysts regress in size or resolve within 12 months of ICL surgery. In our patient, the IPE cyst spontaneously decreased in size, allowing the IOL to return to a more physiological position, thus reducing the hypermetropic refractive error and improving the unaided distance visual acuity over the 2.5 year period.

Our case report suggests that refractive surgery would not be a suitable option because of the changing iris cyst size. Non-permanent methods such as spectacles or contact lenses may be preferred. If a more permanent treatment is sought, then treatment of the IPE cyst rather than refractive surgery may be advisable.

## 4 Conclusion

We report a case of a patient who developed a hyperopic shift following lens replacement surgery due to an IPE cyst. The IPE cyst caused a hyperopic shift due to a substantial increase in size (following lens replacement surgery) that led to displacement of the IOL posteriorly. Over the following months and years, the iris cyst shrunk spontaneously and the unaided visual acuity and refraction improved without any intervention.

## Patient consent

Consent to publish the care report was obtained. This report does not contain any personal information that could lead to the identification of the patient.

## Acknowledgments and disclosures

No funding or grant support.

The following authors have no financial disclosures: KA, ND, VC, MB.

All authors attest that they meet the current ICMJE criteria for Authorship.

## CRediT authorship contribution statement

**Kirupakaran Arun:** Writing – original draft. **Nizar Din:** Writing – review & editing, Supervision. **Miles Stanford:** Writing – review & editing. **Victoria Cosgrove:** Data curation. **Mukhtar Bizrah:** Conceptualization, Data curation, Supervision, Writing – review & editing.

## Declaration of competing interest

The authors declare that they have no known competing financial interests or personal relationships that could have appeared to influence the work reported in this paper.

## References

[1] Georgalas I., Petrou P., Papaconstantinou D., Brouzas D., Koutsandrea C., Kanakis M. (2018). Iris cysts: a comprehensive review on Diagnosis and treatment. Surv Ophthalmol.

[2] Hildreth T., Maino J., Hartong T. (1991 Aug). Primary and secondary iris cysts. J Am Optom Assoc.

[3] Shields J.A. (1981). Primary cysts of the iris. Trans Am Ophthalmol Soc.

[4] Shields C.L., Kancherla S., Patel J. (2012 Feb). Clinical survey of 3680 iris tumors based on patient age at presentation. Ophthalmology.

[5] Dubey Suneeta1, Pegu Julie1, Jain Kanika2 (Oct–Dec 2021). Iris cysts: varied presentations and review of literature. Saudi Journal of Ophthalmology.

[6] Shields J.A., Kline M.W., Augsburger J.J. (1984). Primary Iris cysts: a review of the literature and report of 62 cases. Br J Ophthalmol.

[7] Lois N., Shields C.L., Shields J.A., Mercado G. (1998 Oct). Primary cysts of the iris pigment epithelium. Clinical features and natural course in 234 patients. Ophthalmology.

[8] Köse H.C., Gündüz K., Hoşal M.B. (2020 Mar 5). Iris cysts: clinical features, imaging findings, and treatment results. Turk J Ophthalmol.

[9] Gündüz K., Hoşal B.M., Zilelioğlu G., Günalp İ. (2007). The use of ultrasound biomicroscopy in the evaluation of anterior segment tumors and simulating conditions. Ophthalmologica.

[10] Shields C.L., Kaliki S., Shah S.U., Luo W., Furuta M., Shields J.A. (2012). Iris melanoma: features and prognosis in 317 children and adults. J Am Assoc Pediatr Ophthalmol Strabismus.

[11] Mohite A.A., Prabhu R.V., Ressiniotis T. (2017). Latanoprost induced Iris pigment epithelial and ciliary body cyst formation in hypermetropic eyes. Case Rep Ophthalmol Med.

[12] Kuganasan S., Loo A.V., Subrayan V. (2015). A rare occurrence of epithelial inclusion iris cyst after phacoemulsification. Clin Exp Optom.

[13] Shah A., Rajesh P., Dutta majumder P. (2020). Iris cyst following cataract surgery. Clin Exp Optom.

[14] Wu P.-Y., Wu M.-H., Wu C.-C., Sun C.-C. (2021). Iris cyst after femtosecond laser-assisted cataract surgery: a case report. BMC Ophthalmol.

[15] Zhang X., Chen X., Wang X., Zhou X. (2018 Apr 26). Iridociliary cysts do not impact on posterior phakic intraocular lens implantation for high myopia correction: a prospective cohort study in 1569 eyes. PLoS One.

[16] Shi M., Kong J., Li X. (2015). Observing implantable collamer lens dislocation by panoramic ultrasound biomicroscopy. Eye.

[17] Philip S.S., John D.R., Ninan F., John S.S. (2015 Nov 4). Surgical management of post-traumatic Iris cyst. Open Ophthalmol J.

